# Management of acute exacerbations of COPD in the emergency department and its associations with clinical variables

**DOI:** 10.1007/s11739-024-03592-w

**Published:** 2024-04-11

**Authors:** Ophir Freund, Ariel Melloul, Sabrina Fried, Eyal Kleinhendler, Avraham Unterman, Evgeni Gershman, Avishay Elis, Amir Bar-Shai

**Affiliations:** 1 The Institute of Pulmonary Medicine, Tel-Aviv Sourasky Medical Center, Tel Aviv, Israel; 2 Internal Medicine B, Tel-Aviv Sourasky Medical Center, Tel Aviv, Israel; 3 Faculty of Medicine, Tel Aviv University, Tel Aviv, Israel; 4 Internal Medicine C, Rabin Medical Center, Kfar Saba, Israel

**Keywords:** Chronic obstructive pulmonary disease, Steroids, Short-acting bronchodilators, Diagnosis, Hospitalization

## Abstract

Acute exacerbation of chronic obstructive pulmonary disease (AECOPD) is a common cause for emergency department (ED) visits. Still, large scale studies that assess the management of AECOPD in the ED are limited. Our aim was to evaluate treatment characteristics of AE-COPD in the ED on a national scale. A prospective study as part of the COPD Israeli survey, conducted between 2017 and 2019, in 13 medical centers. Patients hospitalized with AECOPD were included and interviewed. Clinical data related to their ED and hospital stay were collected. 344 patients were included, 38% females, mean age of 70 ± 11 years. Median (IQR) time to first ED treatment was 59 (23–125) minutes and to admission 293 (173–490) minutes. Delayed ED treatment (> 1 h) was associated with older age (*p* = 0.01) and lack of a coded diagnosis of COPD in hospital records (*p* = 0.01). Long ED length-of-stay (> 5 h) was linked with longer hospitalizations (*p* = 0.01). Routine ED care included inhalations of short-acting bronchodilators (246 patients, 72%) and systemic steroids (188 patients, 55%). Receiving routine ED care was associated with its continuation during hospitalization (*p* < 0.001). In multivariate analysis, predictors for patients not receiving routine care were obesity (adjusted odds ratio 0.5, 95% CI 0.3–0.8, *p* = 0.01) and fever (AOR 0.3, 95% CI 0.1–0.6, *p* < 0.01), while oxygen saturation < 91% was an independent predictor for ED routine treatment (AOR 3.6, 95% CI 2.1–6.3, *p* < 0.01). Our findings highlight gaps in the treatment of AECOPD in the ED on a national scale, with specific predictors for their occurrence.

## Introduction

Chronic obstructive pulmonary disease (COPD) is a leading etiology for morbidity and mortality worldwide [1, 2]. It is characterized by acute exacerbations (AECOPD) that have immense implications for poor patient’s outcomes. Different clinical practice guidelines have been developed for the management of AECOPD [1, 3, 4]. The mainstay of treatment includes short-acting bronchodilators (SABD) and systemic steroids, with or without antibiotics based on exacerbation characteristics. Exacerbations lead to hospitalizations, readmissions, disease progression, and decreased quality of life [5, 6]. Therefore, it is necessary to correctly identify exacerbations to initiate treatment early and prevent further complications [7].

AECOPD is a common condition among patients presenting to the emergency department (ED) [8]. It may be the primary reason for ED arrival or the sequela of another acute precipitating event, such as infection or pulmonary embolism [1]. It is estimated that over half of the cases of AECOPD will be discharged from the ED, highlighting the major role of ED physicians in AECOPD care [9, 10]. In addition, it was found that among patients with AECOPD, the median time from ED discharge to first medical follow-up was 13 days, further stressing the importance of proper ED management [9]. Still, while the inpatient management of AECOPD was assessed by multiple large scale studies [11, 12], this is not the case for the ED setting. Prior research focusing on the management of AECOPD in the ED or on its associations with disease outcomes is limited, and mainly includes retrospective or single center studies [13, 14].

For the reasons indicated above, our aim was to evaluate the management of AECOPD in the emergency department on a national scale and its associations with clinical variables and in-hospital care. We hypothesized that a different management of AECOPD in the ED could be associated with changes in care during admission and to specific predictors, which are important for future interventions.

## Methods

The COPD Israeli survey (COPDIS) was a multicenter prospective observational cohort study, conducted at 13 medical centers in Israel, between 2017 and 2019. In general, the COPDIS included subjects hospitalized with AECOPD and aimed to evaluate their care [11]. To be included, subjects had to meet all the following criteria: (1) AECOPD as the main reason for admission, (2) signed informed consent, (3) completion of structured interview, (4) available follow-up from the ED and rest of hospitalization, and (5) available electronic medical records. Subjects were recruited during the mentioned study period, without a predefined target for sample size. AECOPD diagnosis was verified at inclusion by senior physicians. The diagnosis of COPD was based on the diagnostic criteria by the GOLD guidelines [1], which included the presence of prior spirometry with FEV1/FVC < 0.7 and chronic respiratory symptoms as mentioned by patients and/or in prior pulmonologist’s follow-up. Similarly, the diagnosis of AECOPD was based on the GOLD guidelines, and defined as increased dyspnea and/or cough and sputum that worsens less than 14 days, not entirely explained by a different etiology.

The study was approved by each center’s institutional ethical committee and conducted in accordance with the Helsinki declaration. The study was performed in accordance with STROBE guidelines.

### ED and in-hospital variables

ED-related variables included presenting symptoms, oxygen saturation in room air and temperature upon ED arrival, laboratory results, and ED management. ED management comprised of administered medications, investigations performed, and time from hospital arrival to first ED treatment and time to admission. Generally, in the medical centers studied, initial routine treatment for patients presenting with AECOPD includes short-acting bronchodilators (SABD, e.g., short-acting beta-agonists [SABA] or short-acting muscarinic antagonists [SAMA]) and systemic steroids. Accordingly, “routine treatment” was defined for subjects that were given at least one of these treatments at the ED.

Several in-hospital variables were chosen to evaluate their associations with the care at the ED. In-hospital variables included treatments given during hospitalization, hospital length of stay (LOS), and the combined outcome of in-hospital mortality, intubation, or transfer to the intensive care unit (“in-hospital adverse outcome”). In-hospital treatments were defined as COPD-related medications, given during hospitalization for at least 2 days (or until discharge in cases of shorter hospitalization).

### Data collection and analysis

Baseline variables, including demographic and clinical data, were extracted using an electronic case report form (CRF) designed by the COPDIS Steering Committee that was unified between the subjects. Subjects were interviewed by one of the research team. Data from the ED and hospitalization were extracted from electronic medical records during the hospitalization using a predefined database.

Data analysis was performed with SPSS software, version 28.0. Categorical variables were expressed as percentages and continuous variables were presented as median (inter-quartile range), given their non-normal distribution as assessed by Kolmogorov–Smirnov tests. Categorical variables were compared using Chi-square test, and continuous variables were compared using the Mann–Whitney test. Predictors for the administration of routine treatment and for in-hospital adverse outcomes were evaluated using univariate and multivariate logistic regression analyses. The multivariate logistic regression analysis included predictors associated with the outcome in the univariate model, using *p* < 0.2 as a cutoff, and after evaluating their association as clinically relevant by the research team. The method used was backwards elimination with Wald test. Long time from hospital arrival to ED treatment and to hospitalization were defined using median values as cutoffs. The relationship between ambulance transfer and ED LOS was also assessed by Kaplan–Meier analysis.

## Results

344 subjects comprised our study cohort after meeting inclusion criteria. Their median (IQR) age was 71 (63–78), 129 (38%) were females, 290 (84%) had a coded COPD diagnosis in the hospital system upon ED arrival, and 243 (71%) had a prior exacerbation of COPD (Table 1). 140 (41%) subjects arrived via ambulance, and 190 (55%) had oxygen saturation below 91% in room air upon arrival. Treatments for AECOPD at the ED included SABA (44%), SAMA (68%), systemic steroids (55%), inhaled corticosteroids (17%), and antibiotics (36%).

**Table 1:** Characteristics of the study cohort and comparison by administration of routine treatment at the emergency department^a^

Variable	Not given *n* = 88 (%)	Routine treatment *n* = 256 (%)	Total *n* = 344 (%)	*p*
Age, years, median (IQR)	69 (62–79)	71 (63–78)	71 (63–78)	0.98
Female sex	30 (34)	99 (39)	129 (38)	0.44
Charlson score, median (IQR)	5 (3–4)	5 (4–7)	5 (3–7)	0.21
Obese (BMI > 30)	37 (42)	77 (30)	114 (33)	**0.04**
Heart failure	14 (16)	61 (24)	75 (22)	0.12
Atrial fibrillation	8 (9)	26 (10)	34 (10)	0.77
COPD coded diagnosis	72 (82)	218 (85)	290 (84)	0.46
Prior COPD exacerbation	47 (53)	196 (77)	243 (71)	** < 0.01**
Emergency department characteristics				
Ambulance transfer	28 (32)	112 (44)	140 (41)	0.05
Increased dyspnea	75 (85)	238 (93)	313 (91)	**0.03**
Increased cough	40 (45)	155 (61)	195 (57)	**0.01**
Increased mucus	21 (24)	100 (39)	121 (35)	**0.01**
Fever (> 38 °C)	17 (19)	23 (9)	36 (11)	**0.01**
O_2_ saturation < 91%	29 (33)	161 (63)	190 (55)	** < 0.01**
Shift				
Day (07:00–15:00)	43 (49)	125 (51)	168 (51)	0.87
Evening (15:00–23:00)	28 (32)	81 (33)	109 (33)	
Night (23:00–07:00)	16 (18)	39 (16)	55 (17)	
CXR consolidation	11 (13)	21 (8)	32 (9)	0.23
In-hospital variables				
SABD during hospitalization	72 (81)	250 (98)	322 (94)	** < 0.01**
Steroids during hospitalization	69 (78)	238 (93)	307 (89)	** < 0.01**
Hospital length of stay, median (IQR)	4 (3–6)	4 (3–7)	4 (3–6)	0.28
In-hospital adverse outcome^b^	5 (6)	22 (9)	27 (8)	0.38

Bold values indicate *p*-value < 0.05

*COPD* chronic obstructive pulmonary disease, *CXR* chest X-ray, *O*_*2*_ oxygen, *SABD* short-acting bronchodilators

^a^Routine treatment given at the emergency department, including inhalations of short-acting bronchodilators and/or systemic steroids

^b^Adverse events include in-hospital mortality, intubation, or transfer to the intensive care unit

Univariate and multivariate analyses of predictors for the administration of routine treatment at the ED are presented in Tables 1 and 2, respectively. Independent predictors for routine ED treatment were prior COPD exacerbation (adjusted OR 2.38, 95% CI 1.4–4.2, *p* < 0.01), increased mucus production (AOR 2.01, 95% CI 1.1–3.7, *p* = 0.02), and oxygen saturation below 91% (AOR 3.61, 95% CI 2.1–6.3, *p* < 0.01). Independent predictors for omitting routine care in the ED were obesity (AOR 0.48, 95% CI 0.3–0.8, *p* = 0.01) and fever above 38 °C (AOR 0.30, 95% CI 0.1–0.6, *p* < 0.01).

**Table 2:** Predictors for administration of routine treatment at the emergency department^a^

Variable	Univariate	Multivariate	*p*
	OR (95% CI)	Adjusted OR (95% CI)	
Obese (BMI > 30)	0.59 (0.36–0.98)	0.48 (0.28–0.84)	0.01
Prior COPD exacerbation	2.85 (1.71–4.74)	2.38 (1.37–4.15)	< 0.01
Increased mucus	2.05 (1.18–3.55)	2.01 (1.10–3.66)	0.02
Fever (> 38 °C)	0.43 (0.22–0.85)	0.30 (0.14–0.64)	< 0.01
O_2_ saturation < 91%	3.45 (2.07–5.75)	3.61 (2.09–6.25)	< 0.01

*COPD* chronic obstructive pulmonary disease, *O*_*2*_ oxygen

^a^Routine treatment given at the emergency department, including inhalations of short-acting bronchodilators and/or systemic steroids

Initiating routine treatment in the ED was associated with prescribing SABD and systemic steroids during hospitalization (*p* < 0.01 for both, Table 1). In contrast, routine ED treatment was not associated with a change in hospital LOS or with in-hospital adverse outcomes.

The median (IQR) time from hospital arrival to first AECOPD-related treatment in the ED was 59 (23–125) minutes, and the time to hospital admission was 293 (173–490) minutes. Factors associated with delayed ED treatment (> 60 min, Table 3) were older age (*p* = 0.01) and lack of a coded diagnosis of COPD in hospital records (*p* = 0.01). Of note, time to treatment was not associated with the hour of arrival (*p* = 0.95) or the daily shift (*p* = 0.60). Factors associated with long ED stay (> 300 min, Table 3) were prior COPD exacerbation (*p* < 0.01), ambulance transfer to the hospital (*p* < 0.01), and oxygen saturation below 91% (*p* = 0.03). Increased duration in the ED was associated with longer hospital LOS (*p* = 0.01), while delayed treatment was not. A long time to treatment or long ED stay was not associated with in-hospital adverse outcomes.

**Table 3:** Associations between cohort variables and long time from hospital arrival to treatment or to admission ^a^

Variable	Delayed treatment (> 60 min)			Long ED stay (> 5 h)		
	No, *n* = 135 (%)	Yes, *n* = 128 (%)	*p*	No, *n* = 173 (%)	Yes, *n* = 171 (%)	*p*
Age, median (IQR)	70 (62–77)	72 (67–81)	0.01	70 (62–77)	71 (64–80)	0.07
Female sex	51 (38)	51 (40)	0.73	62 (36)	67 (39)	0.52
Charlson score, median (IQR)	5 (3–7)	5 (4–7)	0.68	5 (3–7)	5 (3–7)	0.42
COPD coded diagnosis	121 (90)	100 (78)	**0.01**	150 (87)	140 (82)	0.22
Prior COPD exacerbation	103 (76)	100 (78)	0.72	110 (64)	133 (78)	** < 0.01**
Ambulance transfer	65 (48)	55 (43)	0.40	56 (32)	84 (49)	** < 0.01**
O_2_ sat. < 91%	88 (65)	85 (66)	0.84	97 (56)	116 (68)	**0.03**
Shift (hours)						
Day (07–15)	69 (52)	65 (51)	0.60	85 (53)	83 (49)	0.62
Evening (15–23)	41 (31)	45 (35)		49 (30)	60 (35)	
Night (23–07)	24 (18)	18 (14)		28 (17)	27 (16)	
Hospital LOS, days, median (IQR)	4 (3–7)	4 (3–7)	0.90	4 (3–6)	5 (3–7)	**0.01**

Bold values indicate *p*-value < 0.05

*COPD* chronic obstructive pulmonary disease, *ED* emergency department, *LOS* length of stay, *O*_*2*_ oxygen

^a^Treatments include inhalations of short-acting bronchodilators, antibiotics and/or systemic steroids. If none of these treatments were administered the subject was excluded from the analysis of time to treat

**Fig. 1:**
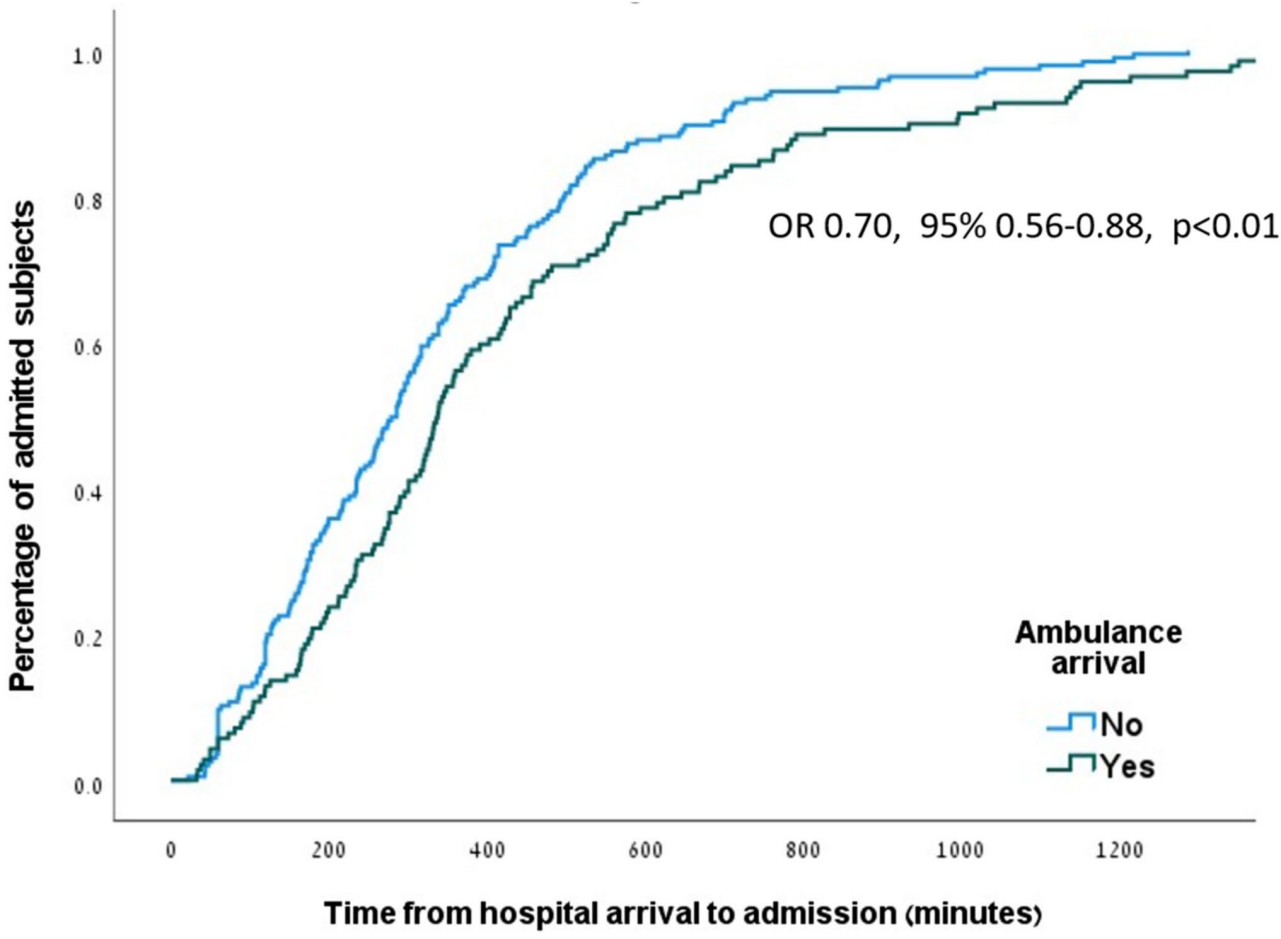
Kaplan–Meier curve of time from hospital arrival to admission, divided to patients with and without ambulance transfer. Comparison between the groups performed by the log-rank test

In a sub-analysis taking into account time to admission, ambulance transfer remained a significant predictor for longer ED LOS (OR 0.703, 95% CI 0.56–0.88, *p* < 0.01, Fig. 1).

Univariate and multivariate analyses of ED-related predictors for in-hospital adverse outcomes appear in Table 4. Independent predictors were female sex (AOR 2.72, 95% CI 1.15–6.49, *p* = 0.02), consolidation in chest X-ray (AOR 3.38, 95% CI 1.13–10.1, *p* = 0.02), high C-reactive protein (CRP > 5 mg/dl, AOR 2.76, 95% CI 1.12–6.75, *p* = 0.03) and acute kidney injury (AOR 3.98, 95% CI 1.57–10.2, *p* < 0.01).

**Table 4:** Patient and emergency department related predictors for in-hospital adverse outcomes^a^

Variable	Univariate		Multivariate	
	OR (95% CI)	*p*	Adjusted OR (95% CI)	*p*
Age, years	1.03 (0.99–1.07)	0.12	–	–
Female sex	2.23 (1.01–4.92)	0.04	2.72 (1.15–6.49)	**0.02**
Charlson score	1.23 (1.05–1.45)	< 0.01	–	–
Prior COPD exacerbation	1.91 (0.70–5.20)	0.20	–	–
Ambulance transfer	2.26 (1.02–5.04)	0.04	2.11 (0.90–4.94)	0.09
Fever (> 38 °C)	0.92 (0.26–3.19)	0.89	–	–
CXR consolidation	3.20 (1.19–8.62)	0.02	3.38 (1.13–10.1)	**0.02**
Leukocytes > 11 10^9^/L	1.29 (0.58–2.88)	0.53	–	–
CRP > 5 mg/dl	3.46 (1.47–8.15)	< 0.01	2.76 (1.12–6.75)	**0.03**
Acute kidney injury^b^	3.67 (1.54–8.75)	< 0.01	3.98 (1.57–10.18)	** < 0.01**
O_2_ saturation < 91%	1.42 (0.63–3.19)	0.40	–	–

Bold values indicate *p*-value < 0.05

*COPD* chronic obstructive pulmonary disease, *CXR* chest X-ray, *CRP* C-reactive protein, *O*_*2*_ oxygen

^a^Adverse events include in-hospital mortality, intubation, or transfer to the intensive care unit. Variables for the multivariate analysis were chosen based on backwards elimination with Wald test

^b^Acute kidney injury is determined as an increase in creatinine of 0.3 mg/dl or more compared to baseline

## Discussion

In the present multicenter study, we evaluate the management of AECOPD in the emergency department, and its associations with in-hospital care. We found independent predictors for not administering routine care in the ED, for delayed treatment in the ED, and for longer ED stay. We also evaluated the association between ED-related variables and in-hospital adverse outcomes.

The main gap we encountered in our cohort was the low rate of routine treatments administered in the ED, which could represent a national issue in Israel. Our results are lower than presented in prior research, with reported rates of 61–91% for SABA and 62–79% for systemic steroids [13–16]. Although not supported by randomized controlled trials, SABDs remain the mainstay of treatment for AECOPD, as recommended by all major clinical guidelines [1, 3, 4]. This is also true for glucocorticoids, especially in cases of hospitalized patients (as is in our study), although research from recent years found that a personalized approach to glucocorticoid treatment was non-inferior [17]. The treatment in the ED was strongly associated with similar treatments during hospitalization, which further highlights the importance of correct treatment during the ED stay. However, it is important to note that our study was not designed to evaluate the effects of administrating routine treatment in the ED, as such study would require a randomized controlled design. In addition, the group that received routine ED treatments had higher rates of variables associated with worse disease (prior exacerbations, worse symptoms, and lower saturation), which could be confounders for the association with in-hospital outcomes.

Proper diagnosis of AECOPD is the key for providing appropriate treatment. Characteristic signs and symptoms of AECOPD would obviously improve the diagnostic rate, while other features could lead physicians to adhere to an incorrect initial impression, known as the anchoring heuristic [18], resulting in diagnostic errors. For example, in a study among patients presenting to the ED with COVID-19 and a second concurrent condition, failing to diagnose the second condition was associated with a larger number of COVID-19-related features [19]. This heuristic could explain why fever at presentation (leading to diagnosis of pneumonia) and obesity (obscuring signs and explaining effort dyspnea) were predictors for missed routine care in the ED. Interventions such as cognitive checklists and electronic triggering systems were shown to facilitate correct diagnosis in similar settings and could help limit diagnostic errors in similar cases [20, 21].

Following a correct diagnosis of AECOPD, different factors might contribute to a lack of routine care in the ED. Issac et al. used qualitative interviews to evaluate factors associated with guideline non-adherence for COPD in the ED [22]. In their study, among the main barriers to guideline implementation were knowledge, professional role clarity, clinical behavior regulation, memory, and attention. For example, the low use of SABA in our study could be partially explained by a fear from its potential pro-arrhythmic effect, while several studies showed a good safety profile in patients without active arrhythmia [23, 24]. Zafar et al. examined COPD care bundle in the ED and found high adherence with recommended treatment [25]. Such bundles can be personalized by local guidelines and improve care without relying on physician’s knowledge and memory.

The median time to first AECOPD-related treatment at the ED was 59 min. A study among patients with asthma exacerbations found shorter times to treatment (17–25 min), depending on the type of medication [26]. In a different study by Ding et al., patients with respiratory chief complaint had the shortest treatment time, although this varied between 45 and 180 min [27]. Adding new tools to the diagnostic process, such as point-of-care ultrasound, may lead to faster treatment in the ED for patients with suspicion of AECOPD [28]. Regarding longer ED LOS, features of severe cases, such as ambulance transfer, prior AECOPD, and low oxygen saturation were its main predictors. Similar features of severe disease were found to be associated with longer ED LOS by Casalino et al. in a study of over 20,000 patients with different conditions [29]. Physicians may want to stabilize a severe patient or see his or her response to treatment before choosing the correct admission setting, resulting in longer ED LOS. We did not find associations between ED LOS and in-hospital outcomes. Whether the mentioned treatment strategy affects patient outcomes and the correct strategy to manage AECOPD at the ED should be the aim of future prospective controlled studies.

Our study has several limitations. First, given its design, only hospitalized patients were included, which limits the generalizability of our results. This could probably lead to a selection bias of more severe cases, and although the effect of ED characteristics on in-hospital variables was one of the aims of this study, it should be taken into account in the interpretation of our findings. Second, enrolment of patients required their informed consent and was at the discretion of the medical teams, leading to possible selection bias. Third, an incorrect diagnosis of AECOPD is possible, as other conditions like heart failure exacerbation could result in similar signs and symptoms. We tried to minimize this limitation by only approaching patients with a main diagnosis of AECOPD and after validation by a senior physician with follow-up during admission. Finally, while ED crowding is known to be a risk factor for time in the ED [30], this variable was beyond the scope of our analysis.

In conclusion, this study adds valuable information on the management of AECOPD in the ED on a national scale. By focusing on different aspects of care, we identified gaps in providing early and correct treatment at the ED, its associations with in-hospital care, and predictors for its occurrence. ED physicians play a major role in the management of AECOPD and we hope to raise awareness of this important topic. Future research should focus on the effect of specific interventions at the ED, with emphasis on improved inpatient care and patient-centered outcomes.

## Data Availability

The authors confirm that the data supporting the findings of this study are available within the article [and/or] its supplementary materials.

## References

[1] 2023 GOLD Report. Global Initiative for Chronic Obstructive Lung Disease-GOLD. https://goldcopd.org/2023-gold-report-2/. Accessed 15–10–2023

[2] Safiri S, Carson-Chahhoud K, Noori M, Nejadghaderi SA, Sullman MJM, Heris JA, Ansarin K, Mansournia MA, Collins GS, Kolahi A-A, Kaufman JS (2022) Burden of chronic obstructive pulmonary disease and its attributable risk factors in 204 countries and territories, 1990–2019: results from the global burden of disease study 2019. BMJ 378:e069679. 10.1136/bmj-2021-069679 35896191 10.1136/bmj-2021-069679 PMC9326843

[3] Wedzicha JA, Miravitlles M, Hurst JR, Calverley PMA, Albert RK, Anzueto A, Criner GJ, Papi A, Rabe KF, Rigau D, Sliwinski P, Tonia T, Vestbo J, Wilson KC, Krishnan JA (2017) Management of COPD Exacerbations: a European respiratory society/American thoracic society guideline. Eur Respir J. 10.1183/13993003.00791-2016 29025888 10.1183/13993003.00711-2017 PMC5678897

[4] National Institute for Health and Care Excellence (NICE). Chronic obstructive pulmonary disease in over 16s: diagnosis and management; NICE, 2019. https://www.nice.org.uk/guidance/NG115. Accessed 01–11–2023 31211541

[5] Seemungal TAR, Donaldson GC, Paul EA, Bestall JC, Jeffries DJ, Wedzicha JA (1998) Effect of exacerbation on quality of life in patients with chronic obstructive pulmonary disease. Am J Respir Crit Care Med 157(5):1418–1422. 10.1164/ajrccm.157.5.9709032 9603117 10.1164/ajrccm.157.5.9709032

[6] Wedzicha JA, Seemungal TA (2007) COPD exacerbations: defining their cause and prevention. Lancet 370(9589):786–796. 10.1016/S0140-6736(07)61382-8 17765528 10.1016/S0140-6736(07)61382-8 PMC7134993

[7] Wilkinson TMA, Donaldson GC, Hurst JR, Seemungal TAR, Wedzicha JA (2004) Early therapy improves outcomes of exacerbations of chronic obstructive pulmonary disease. Am J Respir Crit Care Med 169(12):1298–1303. 10.1164/rccm.200310-1443OC 14990395 10.1164/rccm.200310-1443OC

[8] Hasegawa K, Tsugawa Y, Tsai C-L, Brown DF, Camargo CA (2014) Frequent utilization of the emergency department for acute exacerbation of chronic obstructive pulmonary disease. Respir Res 15(1):40. 10.1186/1465-9921-15-40 24717062 10.1186/1465-9921-15-40 PMC3997196

[9] Rowe BH, Voaklander DC, Marrie TJ, Senthilselvan A, Klassen TP, Rosychuk RJ (2010) Outcomes following chronic obstructive pulmonary disease presentations to emergency departments in alberta: a population-based study. Can Respir J 17:295–300. 10.1155/2010/924978 21165352 10.1155/2010/924978 PMC3006153

[10] Rowe BH, Villa-Roel C, Guttman A, Ross S, Mackey D, Sivilotti MLA, Worster A, Stiell IG, Willis V, Borgundvaag B (2009) Predictors of hospital admission for chronic obstructive pulmonary disease exacerbations in canadian emergency departments. Acad Emerg Med 16(4):316–324. 10.1111/j.1553-2712.2009.00366.x 19298621 10.1111/j.1553-2712.2009.00366.x

[11] Bar-Shai A, Freund O, Ovdat T, Segel MJ, Klempfner R, Elis A (2023) Management of acute COPD exacerbations in the internal medicine departments in Israel–a national survey. Front Med. 10.3389/fmed.2023.1174148 10.3389/fmed.2023.1174148 PMC10483127 37692773

[12] Hurst JR, Quint JK, Stone RA, Silove Y, Youde J, Roberts CM (2020) National clinical audit for hospitalised exacerbations of COPD. ERJ Open Res. 10.1183/23120541.00208-2020 32984418 10.1183/23120541.00208-2020 PMC7502696

[13] Germini F, Veronese G, Marcucci M, Coen D, Ardemagni D, Montano N, Fabbri A, Adinolfi LE, Alvisi A, Azin G, Balloni A, Bandiera G, Barchetti M, Barillari A, Barozzi M, Belloni G, Belotti E, Binetti N, Bonora M, Bruni R, Cacco S, Camisa D, Carbone G, Carpinteri G, Catino L, Cazzaniga M, Cenni P, Chelli V, Cicero L, Cottone CD, Cuccia F, D’Angelo L, Dalmonte F, Daviddi F, Vita AD, Famà F, Fedele M, Fonti C, Frigerio M, Gallingani A, Ghiglione V, Gioffrè-Florio M, Giordano M, Giostra F, Galli MG, Greggi ME, Groff P, Guizzardi S, Lagasio C, Lazzara G, Lubini E, Magni L, Mancarella S, Mangano G, Maragno M, Menabue M, Meoni E, Molinaro F, Morelli A, Moscariello F, Nevola R, Noto P, Pagano A, Paladino F, Pancani R, Petrelli G, Petrino R, Sinno C, Tafa A, Tartaglia S, Taurino C, Treleani M, Villari L, Vitelli A, Zaccagni C, Zaccaro B, Zacchino M (2018) COPD exacerbations in the emergency department: epidemiology and related costs a retrospective cohort multicentre study from the italian society of emergency medicine (SIMEU). Eur J Intern Med 51:74–79. 10.1016/j.ejim.2018.01.010 29371059 10.1016/j.ejim.2018.01.010

[14] Kelly A-M, Van Meer O, Keijzers G, Motiejunaite J, Jones P, Body R, Craig S, Karamercan M, Klim S, Harjola V-P, Verschuren F, Holdgate A, Christ M, Golea A, Graham CA, Capsec J, Barletta C, Garcia-Castrillo L, Kuan WS, Laribi S, on behalf of the AANZDEM and EuroDEM Study Groups (2020) Get with the guidelines: management of chronic obstructive pulmonary disease in emergency departments in Europe and Australasia is sub-optimal. Intern Med J 50(2):200–208. 10.1111/imj.14323 30989793 10.1111/imj.14323

[15] Cydulka RK, Rowe BH, Clark S, Emerman CL, Camargo CA Jr, Investigators, O. B. O. T. M (2003) Emergency department management of acute exacerbations of chronic obstructive pulmonary disease in the elderly: the multicenter airway research collaboration. J Am Geriatr Soc 51(7):908–916. 10.1046/j.1365-2389.2003.51302.x 12834509 10.1046/j.1365-2389.2003.51302.x

[16] Liew CQ, Hsu S-H, Ko C-H, Chou EH, Herrala J, Lu T-C, Wang C-H, Huang C-H, Tsai C-L (2023) Acute exacerbation of chronic obstructive pulmonary disease in united states emergency departments, 2010–2018. BMC Pulm Med 23(1):217. 10.1186/s12890-023-02518-0 37340379 10.1186/s12890-023-02518-0 PMC10283236

[17] Sivapalan P, Lapperre TS, Janner J, Laub RR, Moberg M, Bech CS, Eklöf J, Holm FS, Armbruster K, Sivapalan P, Mosbech C, Ali AKM, Seersholm N, Wilcke JT, Brøndum E, Sonne TP, Rønholt F, Andreassen HF, Ulrik CS, Vestbo J, Jensen J-US (2019) Eosinophil-guided corticosteroid therapy in patients admitted to hospital with COPD Exacerbation (CORTICO-COP): a multicentre, randomised, controlled, open-label. Non-Inferiority Trial Lancet Respir Med 7(8):699–709. 10.1016/S2213-2600(19)30176-6 31122894 10.1016/S2213-2600(19)30176-6

[18] Croskerry P (2003) The importance of cognitive errors in diagnosis and strategies to minimize them. Acad Med 78(8):775 12915363 10.1097/00001888-200308000-00003

[19] Freund O, Azolai L, Sror N, Zeeman I, Kozlovsky T, Greenberg SA, Epstein Weiss T, Bornstein G, Tchebiner JZ, Frydman S (2023) Diagnostic delays among COVID-19 patients with a second concurrent diagnosis. J Hosp Med 18(4):321–328. 10.1002/jhm.13063 36779316 10.1002/jhm.13063

[20] Graber ML, Sorensen AV, Biswas J, Modi V, Wackett A, Johnson S, Lenfestey N, Meyer AND, Singh H (2014) Developing checklists to prevent diagnostic error in emergency room settings. Diagnosis 1(3):223–231. 10.1515/dx-2014-0019 27006889 10.1515/dx-2014-0019 PMC4799784

[21] Murphy DR, Meyer AN, Sittig DF, Meeks DW, Thomas EJ, Singh H (2019) Application of electronic trigger tools to identify targets for improving diagnostic safety. BMJ Qual Saf 28(2):151–159. 10.1136/bmjqs-2018-008086 30291180 10.1136/bmjqs-2018-008086 PMC6365920

[22] Issac H, Taylor M, Moloney C, Lea J (2021) Exploring factors contributing to chronic obstructive pulmonary disease (COPD) guideline non-adherence and potential solutions in the emergency department: interdisciplinary staff perspective. J Multidiscip Healthc 14:767–785. 10.2147/JMDH.S276702 33854328 10.2147/JMDH.S276702 PMC8039430

[23] Wilchesky M, Ernst P, Brophy JM, Platt RW, Suissa S (2012) Bronchodilator use and the risk of arrhythmia in COPD: part 1: saskatchewan cohort study. Chest 142(2):298–304. 10.1378/chest.10-2499 22871755 10.1378/chest.10-2499

[24] Wilchesky M, Ernst P, Brophy JM, Platt RW, Suissa S (2012) Bronchodilator use and the risk of arrhythmia in COPD: part 2: reassessment in the larger quebec cohort. Chest 142(2):305–311. 10.1378/chest.11-1597 22871756 10.1378/chest.11-1597

[25] Zafar MA, Loftus TM, Palmer JP, Phillips M, Ko J, Ward SR, Foertsch M, Dalhover A, Doers ME, Mueller EW, Alessandrini EA, Panos RJ (2020) COPD care bundle in emergency department observation unit reduces emergency department revisits. Respir Care 65(1):1–10. 10.4187/respcare.07088 31882412 10.4187/respcare.07088

[26] Pines JM, Prabhu A, Hilton JA, Hollander JE, Datner EM (2010) The effect of emergency department crowding on length of stay and medication treatment times in discharged patients with acute asthma. Acad Emerg Med 17(8):834–839. 10.1111/j.1553-2712.2010.00780.x 20670320 10.1111/j.1553-2712.2010.00780.x

[27] Ding R, McCarthy ML, Desmond JS, Lee JS, Aronsky D, Zeger SL (2010) Characterizing waiting room time, treatment time, and boarding time in the emergency department using quantile regression. Acad Emerg Med 17(8):813–823. 10.1111/j.1553-2712.2010.00812.x 20670318 10.1111/j.1553-2712.2010.00812.x

[28] Nakao S, Vaillancourt C, Taljaard M, Nemnom M-J, Woo MY, Stiell IG (2020) Evaluating the impact of point-of-care ultrasonography on patients with suspected acute heart failure or chronic obstructive pulmonary disease exacerbation in the emergency department: a prospective observational study. Can J Emerg Med 22(3):342–349. 10.1017/cem.2019.499 10.1017/cem.2019.499 32106899

[29] Casalino E, Wargon M, Peroziello A, Choquet C, Leroy C, Beaune S, Pereira L, Bernard J, Buzzi J-C (2014) Predictive factors for longer length of stay in an emergency department: a prospective multicentre study evaluating the impact of age, patient’s clinical acuity and complexity, and care pathways. Emerg Med J 31(5):361–368. 10.1136/emermed-2012-202155 23449890 10.1136/emermed-2012-202155

[30] Wiler JL, Handel DA, Ginde AA, Aronsky D, Genes NG, Hackman JL, Hilton JA, Hwang U, Kamali M, Pines JM, Powell E, Sattarian M, Fu R (2012) Predictors of patient length of stay in 9 emergency departments. Am J Emerg Med 30(9):1860–1864. 10.1016/j.ajem.2012.03.028 22633732 10.1016/j.ajem.2012.03.028 PMC13477266

